# Discovery of highly active kynureninases for cancer immunotherapy through protein language model

**DOI:** 10.1093/nar/gkae1245

**Published:** 2025-01-07

**Authors:** Hyunuk Eom, Sukhwan Park, Kye Soo Cho, Jihyeon Lee, Hyunbin Kim, Stephanie Kim, Jinsol Yang, Young-Hyun Han, Juyong Lee, Chaok Seok, Myeong Sup Lee, Woon Ju Song, Martin Steinegger

**Affiliations:** Department of Chemistry, Seoul National University, 1 Gwanak-ro, Gwanak-gu, Seoul 08826, Republic of Korea; School of Biological Sciences, Seoul National University, 1 Gwanak-ro, Gwanak-gu, Seoul 08826, Republic of Korea; Galux Inc, 1837 Nambusunhwan-ro, Gwanak-gu, Seoul 08738, Republic of Korea; Department of Chemistry, Seoul National University, 1 Gwanak-ro, Gwanak-gu, Seoul 08826, Republic of Korea; School of Biological Sciences, Seoul National University, 1 Gwanak-ro, Gwanak-gu, Seoul 08826, Republic of Korea; School of Biological Sciences, Seoul National University, 1 Gwanak-ro, Gwanak-gu, Seoul 08826, Republic of Korea; Galux Inc, 1837 Nambusunhwan-ro, Gwanak-gu, Seoul 08738, Republic of Korea; Galux Inc, 1837 Nambusunhwan-ro, Gwanak-gu, Seoul 08738, Republic of Korea; Molecular Medicine and Biopharmaceutical Sciences, Graduate School of Convergence Science and Technology, Seoul National University, 1 Gwanak-ro, Gwanak-gu, Seoul 08826, Republic of Korea; School of Pharmacy, Seoul National University, 1 Gwanak-ro, Gwanak-gu, Seoul 08826, Republic of Korea; Arontier Co., 241 Gangnam-daero, Seocho-gu, Seoul 06735, Republic of Korea; Artificial Intelligence Institute, Seoul National University, 1 Gwanak-ro, Gwanak-gu, Seoul 08826, Republic of Korea; Institute of Molecular Biology and Genetics, Seoul National University, 1 Gwanak-ro, Gwanak-gu, Seoul 08826, Republic of Korea; Department of Chemistry, Seoul National University, 1 Gwanak-ro, Gwanak-gu, Seoul 08826, Republic of Korea; Galux Inc, 1837 Nambusunhwan-ro, Gwanak-gu, Seoul 08738, Republic of Korea; Department of Biomedical Sciences, University of Ulsan College of Medicine, Asan Medical Center, 88 Olympic-ro 43-gil, Songpa-gu, Seoul 05505, Republic of Korea; Galux Inc, 1837 Nambusunhwan-ro, Gwanak-gu, Seoul 08738, Republic of Korea; Department of Chemistry, Seoul National University, 1 Gwanak-ro, Gwanak-gu, Seoul 08826, Republic of Korea; School of Biological Sciences, Seoul National University, 1 Gwanak-ro, Gwanak-gu, Seoul 08826, Republic of Korea; Artificial Intelligence Institute, Seoul National University, 1 Gwanak-ro, Gwanak-gu, Seoul 08826, Republic of Korea; Institute of Molecular Biology and Genetics, Seoul National University, 1 Gwanak-ro, Gwanak-gu, Seoul 08826, Republic of Korea

## Abstract

Tailor-made enzymes empower a wide range of versatile applications, although searching for the desirable enzymes often requires high throughput screening and thus poses significant challenges. In this study, we employed homology searches and protein language models to discover and prioritize enzymes by their kinetic parameters. We aimed to discover kynureninases as a potentially versatile therapeutic enzyme, which hydrolyses L-kynurenine, a potent immunosuppressive metabolite, to overcome the immunosuppressive tumor microenvironment in anticancer therapy. Subsequently, we experimentally validated the efficacy of four top-ranked kynureninases under *in vitro* and *in vivo* conditions. Our findings revealed a catalytically most active one with a nearly twofold increase in turnover number over the prior best and a 3.4-fold reduction in tumor weight in mouse model comparisons. Consequently, our approach holds promise for the targeted quantitative enzyme discovery and selection suitable for specific applications with higher accuracy, significantly broadening the scope of enzyme utilization. A web-executable version of our workflow is available at seekrank.steineggerlab.com and our code is available as free open-source software at github.com/steineggerlab/SeekRank.

## Graphical Abstract

**Graphical Abstract:**
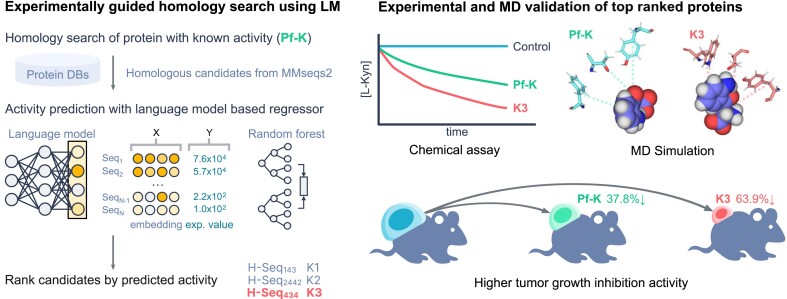

## Introduction

Discovery of enzymes with high catalytic efficiency is both critical and challenging due to the vast search space of potential candidates. Traditional approaches, such as directed evolution or homology searches across extensive protein databases, require the screening of thousands of candidates, making the process time-consuming and resource-intensive (1). Machine learning offers solutions to guide this process by predicting enzyme efficiency (2), thus reducing the number of candidates that need to be experimentally tested. Large protein language models (pLLMs) (3–5), which are pre-trained on vast datasets of amino acid sequences have acquired vast knowledge about both enzyme function (6), structure (7), and binding residues (8), providing a foundational model for developing predictive models if few experimental data points are available (9). By integrating pLLMs with sparse experimental data, we can enhance protein database searches, enabling a targeted approach to prioritize proteins with a high chance of experimental success.

In this regard, kynureninases (KYNase) can be an interesting case to validate our pLLM homology search and to apply the enzymes for therapeutic applications. KYNase is a PLP-dependent enzyme, which hydrolyzes L-kynurenine (L-KYN), which is one of the strong immunosuppressive factors/mediators in the tumor microenvironment (TME) (10). As an AhR ligand, L-KYN induces T cell apoptosis, Treg induction and PD1 upregulation (10,11). Thus, removing the L-KYN in the TME could be an effective anti-cancer therapeutic strategy. Indeed, Triplett *et al.* previously demonstrated that metabolizing L-KYN with exogenous KYNases can enhance the immune response against cancer (12). In particular, KYNase from *Pseudomonas fluorescens* (Pf-K) shows the high catalytic efficiency (*k*_cat_/*K*_M_) compared to the human-encoded KYNase (K0). The administration of Pf-K in combinations with immune checkpoint inhibitors or a cancer vaccine boosted the active CD8^+^ T cell numbers, reduced the tumor growth and increased the mouse survival time by 45% (12). They also conducted iterative saturation mutagenesis and random mutagenesis of K0, leading to the discovery of mutants with a ca. 500-fold increase in the catalytic efficiency with L-KYN or moderate thermal stability (13,14). However, the mutant enzyme showed <75% of the Pf-K activity.

Since Pf-K showed a good therapeutic benefit in mouse model, it is worth of exploring a broader range of KYNases that might offer even greater catalytic efficiency and therapeutic potential. One way to detect more efficient enzymes is by searching for homologous proteins in a large protein database (15) and checking their activity experimentally. However, prioritizing the experiments to execute, while facing thousands of potential homologous candidates remains a major challenge. Although limited, the data provided by Triplett *et al.* (12), which analyzed seven KYNases across the tree of life, combined with the human-specific measurements from the mutant scans patent comprising 159 experimental measures (13), serve as a foundation to prioritize our experiments.

Here we propose a novel computational workflow to address the challenge of prioritizing experiments among thousands of potential homologous candidates when only minimal experimental data are available. Our workflow integrates experimental measurements to rerank homology search results using a pLLM-based regressor trained on-the-fly. We demonstrated the utility of this approach in a case study focused on identifying highly active KYNases for cancer therapy. Additionally, we implemented this workflow as an easy-to-use webserver (seekrank.steineggerlab.com), enabling researchers to conduct similar analyses using their own measures.

## Materials and methods

### Ethical approval and consent to participate

*In vivo* studies were approved by the Institutional Animal Care and Use Committee (IACUC, #2022-12-098) of Asan hospital research institute and that of HELIXITH animal research center (VIC-22-06-004) and performed following the approved procedure.

### Homology search

**Figure 1.**
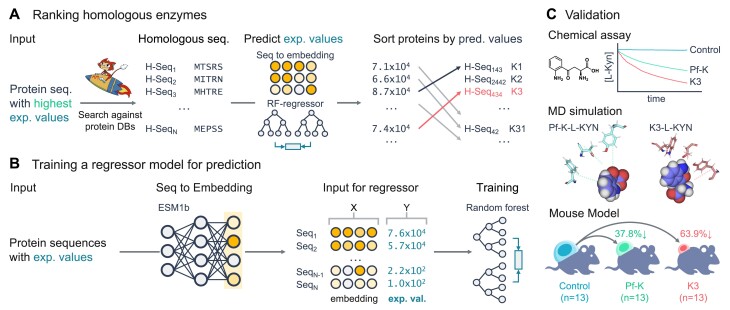
Workflow illustrating the discovery and validation of novel kynureninases for improving cancer immunotherapy. (**A**) Search for homologs of the most catalytically active KYNases (Pf-K), embed them and rank the hits based on their predicted activity. (**B**) Training a regressor model to predict the enzymatic activity from protein using experimentally determined values and pLLM embeddings. (**C**) Validate the four top-ranked homologs in three ways. Catalytic activity assay for monitoring the consumption of L-KYN: the chemical structure and time-dependent consumption of L-KYN. Molecular dynamics (MD) simulations demonstrating the interactions of L-KYN and KYNases. Mouse experiments, depicting the antitumor activity of KYNases.

We searched the KYNase protein sequence of *Pseudomonas* (WP_017531066.1) in a database of cultivated (16) and metagenomic protein sequences from BFD (17) and Metaclust2 (18), a total of 3 144 354 589 protein sequences, using MMseqs2’s (version 3fa46) iterative search (–num-iterations 3 -s 7.5 -c 0.9). The search resulted in 10 195 hits with an *E*-value of <10^–3^ and a bi-directional length overlap of at least 90% similarity. To reduce redundancy, we filtered out sequences with at least 90% sequence identity to another sequence in the set using the filterresult module of MMseqs2, resulting in 5690 remaining sequences. Since the metagenomic sequences do not contain taxonomic labels, we predicted labels using the taxonomy workflow of MMseqs2 (15) by querying the detected sequences against the UniProtKB/TrEMBL + Swiss-Prot (2020_05) database (Figure 1).

### pLLM based regressor to predict *k*_cat_/*K*_M_

To predict the kinetic parameter *k*_cat_/*K*_M_ for enzymes, we implemented a method following the variant prediction workflow proposed in the ESM repository, available at github.com/steineggerlab/SeekRank. Utilizing the ESM-1b model with 670 M parameters pretrained on UniRef50, we collected 159 protein sequences containing measurements of *k*_cat_/*K*_M._ Seven originate from the previously published measurements of sequences from *Homo sapiens*,*Mus musculus*,*Mucilaginibacter Paludis*,*Acinetobacter Baumannii*,*Cyclobacterium marinum*,*Chlamydophila pecorum* and *Pseudomonas fluorescens* (12). The remaining 152 are modified *H. sapiens* sequences with an average 96.5% sequence identity to the reference, extracted from the patent (Supplementary Table S1) (13). For each sequence, we generated ESM-1b sequence embeddings by averaging all residue representations across the whole protein, converting the amino acid sequences into numerical vectors of size 1280.

Subsequently, the dataset was randomly split into training and test sets (80% training and 20% test). We trained three regression models to predict the kinetic parameter, including k-nearest neighbors (KNN), support vector regression (SVR) and random forest regression (RFR). Hyperparameter tuning was conducted using GridSearchCV from scikit-learn, optimizing for the best predictive performance based on the R^2^ metric.

**Figure 2.**
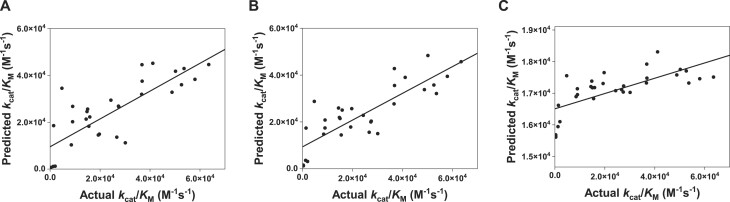
Computational prediction of reactive kynureninases using sequence embeddings. The correlation plots of the experimentally determined *k*_cat_/*K*_M_ values versus the predictions based on (**A**) random forest, (**B**) KNN and (**C**) SVR.

Finally, each model’s performance was evaluated on the test dataset by predicting *k*_cat_/*K*_M_. The Spearman rank correlation coefficient was calculated to assess the models’ predictive performance, yielding 0.802, 0.756 and 0.813 for KNN, SVR and RFR, respectively (Figure 2). Thus, we decided to use the RFR model to rank the proteins detected by the homology search. As a validity check, our RFR model predicted and ranked the enzyme efficiency for all 19 non-wild-type mutations of *H. sapiens* sequence. Notably, 56 of the top 100 mutations were near the enzyme’s active site (position 240–290), confirming the model’s accuracy in identifying key functional regions.

### Validation of the generalizability of the pLLM based regressor

To assess the applicability of the pLLM-based regressor across diverse experimental datasets, we collected 485 sequences from the FPbase (19) and 4351 sequences from the FireProtDB (20). Utilizing the same procedure as pLLM based regressor to predict *k*_cat_/*K*_M_, we predicted *k*_cat_/*K*_M_, trained regressors to estimate brightness and emission peak wavelengths from FPbase, and predicted melting temperature values from FireProtDB.

To evaluate the impact of data size on regressor performance, we conducted random subsampling. For FPbase, we independently subsampled the data 10 times for sizes ranging from 20 to 480, with a step size of 10. For FireProtDB, we subsampled 10 times independently for sizes ranging from 20 to 100 with a step size of 10, and from 100 to 4300 with a step size of 100. We defined the data size with small deviations as the point where 95% confidence interval of the correlation coefficient is <0.1, and the success rate of the process as the ratio of the trials of Spearman rank correlation above 0.5.

### Webserver as Colab Notebook

We have developed a user-friendly Jupyter Notebook, accessible via Google Colaboratory, designed for training customized prediction models using protein sequences and experimental data provided by users in FASTA format (seekrank.steineggerlab.com). Users input protein sequences in FASTA format with experimental measure. These sequences are transformed into embeddings using the ESM-1b model. For prediction, the tool utilizes KNN, SVM and RFR regressors. Here, in contrast to the method above, we optimize the embeddings by checking the predictive performance for each transformer block using a five-fold cross-validation. Post-optimization, we use the backend protein search service for ColabFold (16–18) to search for homologous sequences with the input sequences by searching against the ColabFold database, which is a subset of the 3.2 B proteins, and encodes them into the optimal layer’s embeddings, and then predicts the defined metric, outputting a ranked list of proteins based on these predictions. The protein search service for ColabFold enables users to search extensive, high-quality and metagenomic databases effortlessly, even without access to powerful computing hardware. This is particularly useful when utilizing the tool through platforms like Google Colaboratory.

### Plasmid construction

The putative and previously reported KYNase genes from *Pseudomonas fluorescens* were synthesized after codon optimization for heterologous expression in *Escherichia coli* (Gene Universal). Then, the synthesized gene fragments were inserted into the pET-28b(+) vector using NdeI and XhoI as the cut sites to express N-His_6_-tagged KYNases (Supplementary Table S2).

### Preparation of kynureninases

The custom-ordered plasmids were transformed into *E. coli* BL21(DE3) cells (New England Biolabs). After overnight growth of transformed cells in LB media, the culture (10 ml) was added to TB media (1 l) containing 100 μg/ml of kanamycin in a 4 L-baffled flask. The culture continued at 37°C in an orbital shaker (170 rpm) until the OD_600_ reached 1.0. Before the addition of isopropyl β–*D*-1-thiogalactopyranoside (IPTG) to the final concentrations of 200 μM, the culture was cooled in an ice bath for 10 min. Then, the culture was shaken at the rate of 170 rpm for 16 h at 15°C. Cells were harvested by centrifugation (5000 rpm, 10 min, 4°C), and the cell pellet was stored at −80°C until cell lysis. The cell pellet was thawed in Dulbecco’s phosphate buffered saline (DPBS buffer from Alfa Aesar) containing 1 mM pyridoxal phosphate (PLP from Tokyo Chemical Industry, cofactor) at 4°C, and lysed by microfluidizer (20 000 psi, 2 cycles, LM20 Microfluidics Inc.). After centrifugation (13 000 rpm, 45 min, 4°C) to remove cell debris, the supernatant was filtered through a 0.45 μm syringe filter and loaded to a pre-equilibrated HisTrap HP column (Cytiva) at 4°C using ÄKTA Pure FPLC system (Cytiva). After lysate loading, the column was washed with 8 column volume (CV) of DPBS and the protein was eluted by applying the DPBS containing 300 mM imidazole. The fractions colored in yellow were collected (Supplementary Figure S1) and concentrated using Amicon centrifugal filters (30 kDa cutoff) at 4°C. After the incubation with PLP (1 mM final concentration) at 25°C for 1 h, the protein was applied to size exclusion chromatography (HiLoad Superdex 200 pg column, Cytiva) using DPBS at 4°C. The purity of protein in each fraction was determined using SDS-PAGE (Supplementary Figure S1). For long-term storage, glycerol-containing buffer (20% v/v) was used and stored at −80°C until further use.

### Determination of kinetic parameters

The steady-state specific activity assays were carried out, as reported previously (12,21). In short, we mixed 20 μl of enzyme in DPBS buffer with 80 μl of L-KYN (Sigma-Aldrich) at 37°C. The consumption rate of substrate was monitored at 365 nm using a microplate reader (Synergy H1, Biotek) at 37°C. By applying various concentrations of the substrate (0–700 μM), initial rates were measured. Background reaction rates at high concentration of substrates were subtracted from the observed rates. The kinetic parameters, *k*_cat_ and *k*_cat_/*K*_M_, of enzymes were estimated by fitting into the Michaelis–Menten equation using Origin software.

### System preparation for MD simulations

All calculations were conducted using the X-ray structures of K0 (PDB code 3E9K) and a bacterial KYNase from *Pseudomonas fluorescens* (Pf-K*, PDB code 1QZ9), similar to Pf-K. The missing residues in the original PDB files were modeled with the MODELLER program (22). The PyMOL program was utilized to build the symmetric protein conformation. Molecular docking of L-KYN were conducted using GalaxyDock3 (23). For ligand docking, the substrate binding site was assigned based on the inhibitor site in the X-ray structures. For each enzyme, the initial complex structure for MD simulation had the lowest docking energy among the conformations where the distance between the amine group of substrates and the internal aldimine linkage (C4A) of PLP is <3.5 Å. This selection was crucial as the enzyme reaction initiates with the transamination of the C4A atom of PLP and K247 (amine). The 3D-structure of K3 was modelled with AlphaFold2 (24). The energy-minimized structure of Pf-K*/L-KYN served as a template for Alphafold2 modeling.

### Molecular dynamics simulations

All-atom MD simulations were performed using the *PMEMD.cuda* module of AMBER 20 package (25) with employing the ff14SB force field (26). Each of three enzyme-substrate combinations, K0/L-KYN, Pf-K*/L-KYN and K3/L-KYN was solvated in a cubic box using TIP3P water molecules (27). Counter ions, Na^+^ and Cl^-^, were added to neutralize with a concentration of 150 mM. The system underwent energy minimization, including 500 steps of steepest descent minimization followed by 500 steps of conjugate gradient minimization. This was followed by the second energy minimization, consisting of 1000 steps of steepest descent minimization followed by 1500 steps of conjugate gradient minimization. The equilibration process involved three steps in two phases. In the first equilibration step, the system was heated from 0 to 310 K over 100 ps with weak harmonic restraints of 10.0 kcal/mol/Å^2^ applied to entire simulation system in the NVT ensemble. The second equilibration step applied the same amount of harmonic restraints to the backbone atoms of the enzyme-substrate complexes for 100 ps. In the third equilibration step, 1.0 kcal/mol/Å^2^ of harmonic restraints were applied to Cα atoms of the complex system for 100 ps. The final equilibration phase was performed in the NPT ensemble by Langevin thermostat (28) and Berendsen barostat (29) for 4 ns without restraints. Subsequently, a production simulation was conducted for 200 ns, which was repeated 10 times for each system. A cutoff of 10 Å was employed for non-bonded interactions. Long-range electrostatic forces were calculated with the Particle Mesh Ewald method (30).

### Trajectory analysis

The MD trajectories were analyzed using *CPPTRAJ* (31) and in-house Python scripts. An atomic distance fluctuation was calculated using the distances between N of aniline ring of L-KYN and side-chain polar O of binding pocket residues. For hydrophobic residues such as Phe, CB atom was selected. Each snapshot was taken every 1 ns for 200 ns simulation. Cavity volume analysis was performed by CAVER 3.0 built in PyMol (32).

### pH-dependent activity assay

Specific activities were determined by measuring consumption rates of L-KYN (700 μM) at 25°C in pH 5.0–8.0 buffers; 50 mM citric acid (pH 5.0–6.0); and potassium phosphate (pH 6.5–8.0). Before measuring the specific activity, enzymes were pre-incubated in each buffer for 10 min at 25°C.

### Preparation of PEGylated kynureninases

The recombinant proteins were expressed and purified as described above, and phase separation method was applied to remove endotoxin as described previously (33). In short, Triton™ X-114 (Sigma-Aldrich, 1% v/v) was directly added to the protein solution and mixed by pipetting at 4°C. After incubation for 10 min, the samples were warmed at 37°C for 5 min to allow phase separation. Samples were then centrifuged (13 000 rpm, 1 min, 37°C), and the upper yellow aqueous phase was collected using a micropipette. This procedure was repeated twice to ensure that low endotoxin level for all the samples. Before PEGylation, the resulting samples were incubated with 1 mM PLP at 25°C for 1 h. Then, 100-fold molar excess methoxy-PEG-CO(CH_2_)_2_COO-NHS to the protein (5 kDa, NOF America Corporation) was directly added as powder and incubated for 1 h at 25°C. The unreacted PEGylation reagents were removed by centrifugal filters at 4°C. The endotoxin level was determined using a chromogenic endotoxin quantification kit (*Limulus* amebocyte lysate from Thermo Fisher Scientific), showing <0.2 EU/ml for all samples. The purity and the degree of PEGylation were determined using SDS-PAGE and size exclusion chromatography (HiLoad Superdex 200 pg column, Cytiva), respectively (Supplementary Figure S2).

### Thermal stability of kynureninase activity

Non-PEGylated and PEGylated proteins (10 μM) were incubated at 37°C using a thermal cycler (Bio-Rad Laboratories). After 12 and 24 h, specific activities were measured in the presence of L-KYN (700 μM) in DPBS at 37°C as described above. Alternatively, we measured the CD spectra of KYNases under various temperatures. Additionally, the stability of PEGylated KYNases in human serum was determined by measuring the residual catalytic activity of PEGylated enzymes after incubation in the presence of 98% human serum (Sigma-Aldrich) at 37°C for 6, 12 and 24 h.

The CD spectra were recorded within the far-UV range (190–250 nm) at a protein concentration of 1 μM in 10 mM sodium phosphate buffer, pH 7.4 at 25°C, in a 1 mm path length quartz cuvette (Jasco) using a spectropolarimeter (J-815, Jasco). For each sample, 10 accumulated scans were averaged and then converted into mean residue ellipticity. The thermal denaturation of non-PEGylated and PEGylated proteins (5 μM) was measured by following the change in ellipticity at 222 nm as a function of temperature increasing from 25 to 95°C. The melting temperatures were determined by fitting the data to the equation of a sigmoidal function.

### Cell lines and mice

The mouse B16-F10 melanoma and CT26 colon cancer cell lines were purchased from the ATCC (American Type Culture Collection, Manassas, Virginia, USA, #CRL-6475). Cell line was maintained in DMEM (Dulbecco’s Modified Eagle’s Medium, Corning, New York, USA #10-013-CV) with 10% FBS (Fetal Bovine Serum, Corning, New York, USA #35-015-CV) and 1% penicillin/streptomycin (Thermo, Waltham, MA, USA, #10378016) under the condition of 37°C in a 5% CO_2_ humified incubator. C57BL/6 inbred mice were purchased from Jackson lab (Bar Harbor, Maine, USA) and BALB/c mice were from Raonbio (Yongin, South Korea). *In vivo* studies were approved by the Institutional Animal Care and Use Committee (IACUC, #2022-12-098) of Asan hospital research institute and that of HELIXITH animal research center (VIC-22-06-004) and performed following the approved procedure.

### *In vitro* T cell activation

CD8^+^ T cells were purified from spleen of C57BL/6J mouse using EasySepTM Mouse CD8^+^ T cell isolation kit (STEMCELL, Vancouver, Canada, #19853) by using an EasySep™ magnet (STEMCELL, Vancouver, Canada, #18000) following the protocols provided by the supplier. Purified CD 8^+^ T Cells were plated on a round bottom 96well plate precoated with 5 μg/ml of mAnti-CD3 (clone:145-2C11, ebioscienceTM, CA, USA, #16-0031-96) in a T cell activation media consisting of RPMI1640 (#22400-071), 10% FBS (#26140-087), β-mercaptoethanol (55 μM, #21985–023), 1× GlutaMax (#35050-061), 100 mM penicillin/streptomycin (#10378-016) and 200 mM sodium pyruvate (#11360-070), that purchased from Gibco (Waltham, Massachusetts, USA), mAnti-CD28 (clone:37.51, ebioscienceTM, CA, USA, #16-0281-86, 2 μg/ml) and rhIL-2 (PEPROTECH, NJ, Cranbury, USA, #200-02, 400 U/ml), in the presence or absence of L-KYN (Sigma, MA, USA, #K8625-100. 1 mM) and of Pf-K-PEG or K3-PEG (0.02 uM or 0.05 μM). The aggregated T cell group size, indicative of T cell proliferation level, was observed under the microscope (ZEISS primovert, Oberkochen, Germany, #491206-0001-000) on day 5 after activation. Additionally, The cell number was counted with hemocytomter (MARIENFELD, Lauda-Königshofen, Germany, # 0640010) on day 7 after activation.

### Syngeneic implant tumor model

To establish a syngeneic implant tumor model, B16-F10 melanoma and CT26 colon cancer cell lines were implanted subcutaneously into the flank of female C57BL/6 mice (6 wks) and BALB/c mice, respectively. When the tumor volume reaches ∼50mm^3^(on 7days post injection), mice were classified equally and randomly into three groups (*n* = 13). Pf-K-PEG [dosage: 20 mg/kg] and K3-PEG [dosage: 20 mg/kg] via peritumoral injections were administered to the mice a total of three times twice a week. The tumor volume was measured twice a week, calculated according to the common formula: *V* = (Length × Width^2^) × 1/2, T/C ratio(%) was measured as the tumor volume in the control group to the tumor volume in the treated group. TGI(%) was measured as the tumor volume in the control group to the tumor volume in the treated group according to formula: (V_c_ – V_t_)/ V_c_ × 100, V_c_ = Tumor volume in the control group at the time of tumor extraction / end point. V_t_ = Tumor volume in the treatment group at the time of tumor extraction/end point.

## Results

### Detection of kynureninases in protein databases

To find candidate KYNases, we propose to utilize homology search and pLLMs (3) to find and rank the catalytic activities of KYNases (Figure 1A). We searched with MMseqs2 (15) for sequences similar to Pf-K, across database containing 3.2 billion protein sequences (16–18) extracted from known species and uncultivated sources. We identified 10 195 sequences that closely match Pf-K over at least 90% of their lengths and meet a strict *E*-value threshold of <0.001. To remove redundant sequences, we grouped them based on 90% sequence identity, resulting in a set of 5690 sequences. We then used MMseqs2 taxonomy (15) to assign a likely species origin to each sequence. The taxonomical distribution of these sequences across different species is shown in Supplementary Figure S3.

### Prediction of reactive kynureninases using sequence embeddings

For each detected protein sequence, we predicted the kinetic parameters of catalytic efficiency (*k*_cat_/*K*_M_), using a predictor based on embeddings generated by the ESM1 pre-trained language model (3). Our predictor transforms protein sequences into fixed-size vector-space embeddings using language models, then predicts *k*_cat_/*K*_M_ via a RFR model. Trained on an 80/20 split of 159 experimentally measured sequences (Figure 1B), seven from the previously published measurements and the remaining 152 extracted from a patent, this model achieves a Spearman correlation of 0.813, outperforming that of 0.802 and 0.756 for KNN and SVR, respectively, denoting high predictive accuracy (12,13) (Figure 2). Furthermore, we extended our analysis to other language models such as ESM2 (4) and ProtT5 (5). The RFR consistently showed superior performance in a fivefold cross-validation. Notably, ESM1 retained the highest Spearman correlation score of 0.7411, followed closely by ProtT5 and ESM2, which scored 0.7232 and 0.7196, respectively.

To evaluate the generalizability of the method across different types of experimental data, we applied it to two distinct databases: FPbase, which contains data on fluorescent proteins, and FireProtDB, which includes protein stability data. Specifically, we used the method to analyze three experimental values—brightness and emission peak wavelengths from FPbase, and melting temperature values from FireProtDB. The Spearman rank correlation was calculated for KNN, SVR and RFR within each category. The resulting correlations were 0.70, 0.58 and 0.72 for brightness; 0.88, 0.85 and 0.87 for emission peak wavelengths; and 0.82, 0.81 and 0.84 for melting temperature values, respectively (Supplementary Figure S4A).

Additionally, we assessed the minimum training data size required to achieve minimal variation in Spearman correlation, and the success rate of the process by the data size, which is calculated as the ratio of results showing Spearman rank correlation >0.5. The results showed that 200, 200 and 300 data points were necessary for the three datasets, respectively, to achieve this small variation using the RFR model, and the success rate gradually increased with the data size (Supplementary Figure S4B and D).

Furthermore, we compared our approach to UniKP (34), a specialized neural network designed to predict the catalytic efficiency *k*_cat_/*K*_M_ of enzymes. UniKP showed a Spearman rank correlation of −0.484 on our datasets, which is lower than the correlation achieved by our method, indicating superior performance of our RFR.

We then ranked the 5690 protein sequences detected by MMseqs2 using RFR. The 10 highest ranked sequences were all prokaryotic, 4 were predicted to be from the *Pseudomonas* genus and the remaining 6 were from various *Proteobacteria*. As a general trend, our predictor predicts higher *k*_cat_/*K*_M_ for sequences from bacteria than from eukaryotes, which is in line with what has been previously known (see Supplementary Table S3) (12). Next, we chose four high-ranking sequences for validation. The highest-ranking was a KYNase from the *Pseudomonas* genus (K1) that has a 70.67% sequence identity to Pf-K; the second best was from *Pseudomonas* sp. (K2), and third *Bordetella genomosp*. (K3) with a 64.42% sequence identity. For further comparison, we selected an additional sequence from *Rheinheimera* sp., ranked 31st (K31), for heterologous expression in *E. coli* (Supplementary Figure S1 and Supplementary Table S2).

To determine whether the regressor primarily selected proteins that were most similar in sequence to Pf-K, the enzyme with the highest *k*_cat_/*K*_M_ value in the training data, we sorted the identified proteins in two ways: by sequence identity to Pf-K and by predicted *k*_cat_/*K*_M_ values. The results showed that sequence identity to Pf-K fluctuated significantly among the top 50 hits based on predicted *k*_cat_/*K*_M_ values, ranging from 0.398 to 0.877. This variability suggests that the regressor is considering factors beyond just sequence identity to Pf-K (Supplementary Figure S5).

### Biochemical characterizations of putative kynureninases

**Figure 3.**
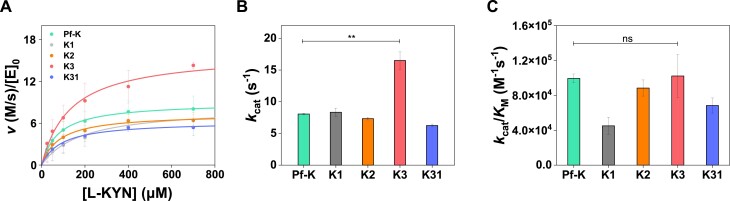
Kinetic analysis of putative kynureninases. (**A**) Michaelis–Menten kinetic analysis. (**B**) Determined turnover rates (*k*_cat_) and (**C**) catalytic efficiencies (*k*_cat_/*K*_M_). Statistical significance was verified with *t*test (two-tailed), *P* value (* *P* ≤ 0.05, ** *P* ≤ 0.01, ns; not significant).

All four putative KYNases we selected show comparable or even substantially higher kinetic parameters than Pf-K (12,21) (Figure 3A–C and Supplementary Table S4); in particular, K3 exhibits a ∼2-fold higher turnover rate (*k*_cat_) while *k*_cat_/*K*_M_ value is comparable with that of Pf-K (Figure 3B and C, and Supplementary Figure S6). Despite the inconsistency between the predicted order in *k*_cat_/*K*_M_ and the experimental results, these findings underscore the effectiveness of our predictor in identifying enzymes with high catalytic efficiency. Furthermore, these results also indicate that K3 may be a promising candidate for therapeutic applications.

### Investigating molecular mechanism of K3 and Pf-K

To elucidate the molecular mechanism underlying the enhanced catalytic activity of K3, we performed MD simulations for 200 ns on K0, K3 and Pf-K*, each bound to their respective substrates. We hypothesized that a stable binding conformation between the pocket residues and the substrates may facilitate catalytic activity since the substrate-binding is the prerequisite for catalytic activities. Throughout the MD simulations, the inter-atomic distances between the pocket residues of K3 and L-KYN remained stable (Supplementary Figure S7A). Specifically, the aniline ring of L-KYN consistently maintained stable hydrophobic interactions with Y226 and W257 over the entire MD trajectory. In contrast, the corresponding interactions between L-KYN and the pocket residues in Pf-K* and K0—namely Y226, F257 and T282—were more flexible and unstable. In the MD simulations of Pf-K* and K0, the distances between the pocket residues and L-KYN increased from their initial conformations (Supplementary Figure S7B). This difference could be attributable to variations in cavity volume. The binding site of K3 exhibits a denser arrangement compared to that of Pf-K*, with a cavity volume 0.3−0.8 Å³ smaller than that of Pf-K* (Supplementary Table S5). In summary, these MD simulation results imply that the mutation from F257 to W257 reduces the volume of the binding pocket, leading to a more stable reactive geometry. This mutation may promote the formation of a stable conformation of L-KYN, thereby enhancing the catalytic efficiency of K3.

Our results are consistent with a recent parallel evolution study of K0 (35). In this study, two K0 mutants, HsKYNase_93D9 and HsKYNase_66, were evolved, each exhibiting different enzymatic activities. HsKYNase_66 displayed enhanced activity toward KYN but reduced activity toward OH-KYN, while HsKYNase_93D9 showed enhanced activities toward both KYN and OH-KYN. In MD simulations, HsKYNase_66 formed more stable interactions with KYN but exhibited reduced interactions with OH-KYN compared to K0. Similarly, HsKYNase_93D9 demonstrated more stable interactions with both KYN and OH-KYN. Taken together with our results, these findings suggest that the stability of key interactions between active site residues and substrates, as observed in MD simulations, may offer valuable insights into the changes in enzymatic activities.

### The pH-dependent activity and stability of kynureninases

**Figure 4.**
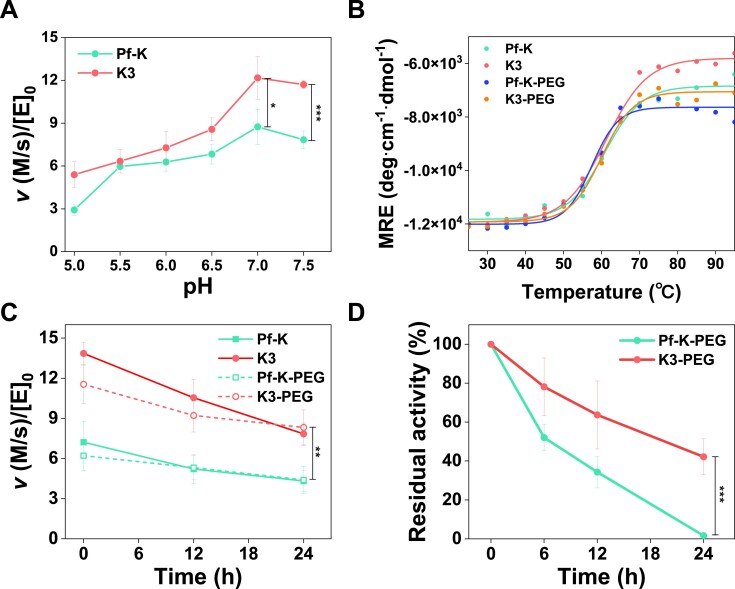
Biochemical characterization of kynureninases. (**A**) pH-dependent activity of Pf-K and K3. (**B**) Temperature-dependent CD spectral changes. The thermal denaturation of non-PEGylated and PEGylated proteins (5 μM) was measured by following the change in ellipticity at 222 nm as a function of temperature increasing from 25 to 95°C. (**C**) Catalytic activities measured after incubation of the enzymes in DPBS at 37°C for the indicated time. (**D**) Residual activities of PEGylated proteins after incubation in 98% human serum at 37°C for the indicated time. Statistical significance was verified for *t*test (two-tailed), *P* value (* *P* ≤ 0.05, ** *P* ≤ 0.01, *** *P*≤ 0.001).

The pH-dependent activity and thermal stability of enzymes can be critical in cancer treatments (36). Thus, we first assessed the steady-state catalytic activity of K3 under various pH conditions. Although K3 shows lower catalytic activity at acidic pH conditions found in tumors, it retained sufficiently higher activities than Pf-K (Figure 4A). The thermal stability of K3 was also comparable with that of Pf-K; the melting temperature (*T*_m_) of Pf-K and K3 were 60.8 and 61.1°C, respectively (Figure 4B). PEGylation was introduced to the enzymes for extending their half-lives for *in vivo* application. K3 and Pf-K were PEGylated at comparable extents (Supplementary Figure S2), and they showed no detectible perturbation in the secondary structures (Supplementary Figure S8). The PEGylated enzymes, K3-PEG and Pf-K-PEG, retained catalytic activity and stability; 86% and 83% relative to their non-PEGylated ones, and melting temperature of 57.4 and 60.0°C (Figure 4B). However, the catalytic activity of K3 remained superior to that of Pf-K after 24 h enzyme incubation in DPBS buffer at 37°C (Figure 4C), Interestingly, K3-PEG exhibited 26-fold higher residual activity than Pf-K-PEG after incubation in the presence of 98% human serum at 37 °C for 24 h (Figure 4D). Although the substantially higher stability and residual catalytic activity of K3-PEG under physiological conditions were not specifically targeted or expected, these properties would greatly benefit the T-cell and physiological mouse experiments.

### Kynureninase K3 reverse L-KYN-induced T-cell proliferation inhibition better

**Figure 5.**
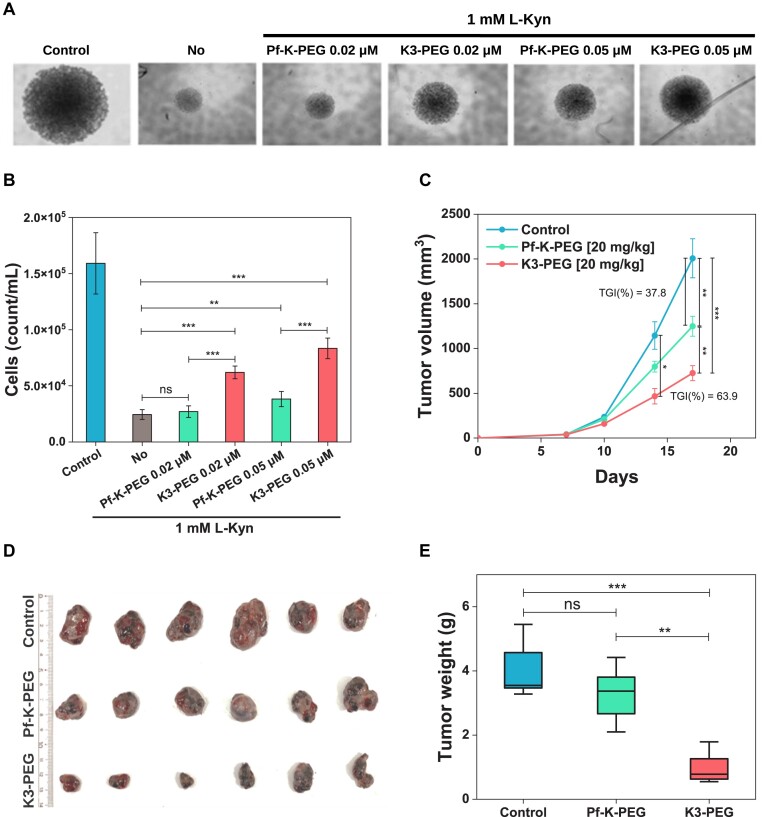
Antitumor effects of Pf-K-PEG and K3-PEG *in vitro* and *in vivo*. (**A**) Reversal of L-KYN-induced inhibition of T cell activation/proliferation *in vitro*. (**B**) CD8^+^ T cell counts measured after T cell stimulation *in vitro* (at day 7). (**C**) Tumor growth curve of B16-F10 bearing mice (C57BL/6) that were treated with peritumoral injection of control (PBS, *n* = 13), Pf-K-PEG (20 mg/kg, *n* = 13) and K3-PEG (20 mg/kg, *n* = 13). (**D**) Representative tumor images at the end of the monitoring. (**E**) Tumor weight after isolation at the end of monitoring. Statistical significance was verified for *t*-test (two-tailed), *P* value (*P ≤ 0.05, **P ≤ 0.01, ***P ≤ 0.0001). Error bars represents the mean ± SEM.

To investigate whether PEGylated KYNases can reverse L-KYN-induced inhibition of the activation/proliferation of CD8^+^ T cells, Pf-K-PEG and K3-PEG were treated in the presence of L-KYN after T cell activation. The size of T cell groups formed after T cell proliferation was reversed more strongly in the culture treated with K3-PEG than Pf-K-PEG (Figure 5A). Consistently, the cell counts showed the similar pattern to the aggregate T cell group size (Figure 5B) (*P*-value = 0.039, on day 7). This result indicates that K3-PEG is functionally more active than Pf-K-PEG in the biologically relevant context.

### K3-PEG inhibits tumor growth more effectively than Pf-K-PEG *in vivo*

To evaluate the anti-tumor efficacy of Pf-K-PEG and K3-PEG *in vivo*, we employed a syngeneic mouse melanoma implant tumor model and administrated PEG-KYNase peritumorally as previously reported (12). Both K3-PEG and Pf-K-PEG samples were prepared after endotoxin removal and further purification, as described in the ‘Materials and methods’ section. As expected, K3-PEG-treated group showed much stronger tumor growth inhibition [TGI(%) = 63.9, *P*-value = 0.00008] than that of Pf-K-PEG [TGI(%) = 37.8, *P*-value = 0.0067] (Figure 5C–E and Supplementary Figure S9). However, the KYNase-treated mice showed no significant body weight change compared to the control (Supplementary Figure S10). Additionally, *in vivo* anti-tumor efficacy test using colon cancer mouse model showed similar result (Supplementary Figure S11). These results together indicate that K3-PEG is an effective anti-cancer enzyme, validating our enzyme search method.

## Discussion

IDO1 and/or TDO initiate the production of L-KYN, a major immunosuppressive metabolite, by breaking down tryptophan (Trp). Therefore, small molecule IDO/TDO inhibitors were developed to overcome the L-KYN-indcued strong immunosuppressive TME (37). However, these approach were not successful in clinical trials (37). KYNases have several pharmacological advantages over small molecules. First, the complete blockade of Trp catabolism by IDO1 and TDO dual inhibitors may induce serious side effects such as high levels of Trp-induced neurological effects and blockade of synthesis of downstream neuroprotective compounds (12). In contrast, KYNase may not greatly perturb the Trp catabolism by removing the metabolites downstream. Secondly, small molecule inhibitors of enzymes frequently result in the acquisition of drug-resistant mutations (38), while KYNase is not relevant for such a mechanism since it degrades the immunosuppressive metabolite itself.

All four selected enzymes showed relatively high catalytic activity. In particular, K3 showed 2-fold better enzymatic activity than the published best KYNase, Pf-K and stronger tumor growth inhibition in animal models. Furthermore, the higher stability and remained activity of K3-PEG in human serum makes it a more potent therapeutic enzyme candidate than Pf-K-PEG. Since the KYNase is originated from bacteria, K3 administration could cause immunogenicity risk *in vivo*, although bacterial enzymes, such as asparaginases, have been FDA-approved and used for certain cancer treatment (39). PEGylation may help shield KYNase from immune recognition (40); thus PEG-K3 is open to further development for anticancer drug.

Our method succeeded in finding the better-performing KYNase, K3, than Pf-K. We showed that the method is generalizable for multiple datasets including FPbase and FireProt. The correlation between RFR-predicted KYNase activities and actual activity was high enough to find novel KYNase with high *k*_cat_/*K*_M_ even with the high average pairwise sequence identity of 0.926 within training set (Supplementary Table S1). However, the size of the training set, 159 was not enough to fully train regressors to work consistently with other regression models. We noticed a subtle difference in the Spearman rank correlations between the RFR and the KNN regressor. When comparing the top hits identified by the RFR (K1, K2, K3 and K31) to those ranked by the KNN regressor, we observed significant discrepancies, with K1, K2, K3 and K31 being ranked 349th, 320th, 1324th and 423rd, respectively. Also, when we calculated the Spearman rank correlation between the predicted *k*_cat_/*K*_M_ values from both methods, a moderate correlation of 0.593 was observed, indicating that the models may have learned different features independently. Although we did not conduct further experiments on the top hits from the KNN regressor, it would be worthwhile to explore these results in future studies.

Further analysis of the Spearman rank correlation as a function of data size revealed that the correlation between the RFR and KNN regressor increases as the data size grows (Supplementary Figure S4C). This suggests that the moderate correlation observed in our results may be due to the relatively small dataset used. We also collected non-redundant sequences with the same enzyme commission (EC) number, functional parameters and reactants from BRENDA (41). This resulted in up to 81 non-redundant sequences for each unique EC number, functional parameter and reactant, generating five datasets containing >50 sequences. When we trained a RFR using the same approach, the Spearman’s rank correlation coefficients on the test set were ranging from −0.07 to 0.65, which are lower than those observed with FireProtDB and FPbase. This discrepancy could have been caused by the small size of dataset we could gather from the BRENDA datasets, than those in the other databases. These findings, consistent with the results from the subset sampling experiments, highlight the crucial role of data size in model performance, as larger datasets allow for more accurate predictions and better capture the relationship between mutations and functional parameters.

In addition to the size of train data size, the skewness or imbalance can impact the regression performance. The FPbase brightness dataset we used in our evaluation was more skewed compared to others (FPbase brightness: 1.44, emission: 0.56, FireProtDB: 0.116, based on Fisher’s moment coefficient of skewness). The correlation coefficient from the RFR trained on this skewed dataset was 0.72, which, while strongly positive, was relatively lower than that from the other datasets (0.87 and 0.84). This difference may be attributed to the skewness of the training values. To improve training performance on skewed or imbalanced fitness scores, it is recommended to apply appropriate transformations to the training data before fitting regressors.

While SeekRank leverages minimal task-specific training data from experiments to predict protein functionality, further improvements could be achieved by integrating complementary strategies. Structure-informed language models, such as ESM-IF1, can reduce reliance on task-specific training datasets (42), and mixtures of models can allow to explore broader fitness landscape (43). Although SeekRank currently focuses on single-property optimization, optimizing multiple properties simultaneously is often important in enzyme design. Future research could focus on reducing training data dependency, expanding coverage of the fitness landscape and extending the method to handle multiple objectives.

## Conclusion

Our work demonstrated that homology search combined with pLLMs can detect and prioritize highly catalytically active therapeutic enzymes even when only little labelled training data are available. Since our approach can be applied to any kind of enzymes, we believe that our work will significantly broaden the scope of enzyme utilization in the industrial and medical purposes. As the best catalytic KYNase identified in this study could be developed as a new anticancer drug, more potential therapeutic enzymes are expected to be identified using our approach in future.

## Supplementary Material

gkae1245_Supplemental_File

## Data Availability

We provide the data used to perform the analysis at 10.5281/zenodo.10517668. We also build an executable version of the workflow, which is available at seekrank.steineggerlab.com. We deposited the code at https://github.com/steineggerlab/SeekRank and https://doi.org/10.5281/zenodo.14263531.
