# Supplementary material for: Discovery of highly active kynureninases for cancer immunotherapy through protein language model
Source: Nucleic Acids Res. 2025 Jan 7;53(1):gkae1245. doi: 10.1093/nar/gkae1245 (PMC11704957; doi:10.1093/nar/gkae1245)
Supplement: gkae1245_Supplemental_File [file gkae1245_supplemental_file.docx]

**Supplementary Data for**

Discovery of Highly Active Kynureninases for Cancer Immunotherapy through Protein Language Model

Hyunuk Eom^4^, Kye Soo Cho^6^, Jihyeon Lee^4^, Stephanie Kim^1^, Sukhwan Park^1^, Hyunbin Kim^1^, Jinsol Yang^6^, Young-Hyun Han^6^, Juyong Lee^7,8,9^, Chaok Seok^2,3,4,6^, Myeong Sup Lee^5,6*^, Woon Ju Song^4*^, Martin Steinegger^1,2,3*^

^1^School of Biological Sciences, Seoul National University, Seoul, 08826, Republic of Korea

^2^Artificial Intelligence Institute, Seoul National University, Seoul, 08826, Republic of Korea

^3^Institute of Molecular Biology and Genetics, Seoul National University, Seoul, 08826, Republic of Korea

^4^Department of Chemistry, Seoul National University, Seoul 08826, Republic of Korea

^5^Department of Biomedical Sciences, University of Ulsan College of Medicine, Asan Medical Center, Seoul 05505, Republic of Korea

^6^Galux Inc, Gwanak-gu, Seoul 08738, Republic of Korea

^7^Molecular Medicine and Biopharmaceutical Sciences, Graduate School of Convergence Science and Technology, Seoul National University, Seoul 08826, Republic of Korea

^8^School of Pharmacy, Seoul National University, Seoul 08826, Republic of Korea

^9^Arontier Co., Seoul 06735, Republic of Korea

^*^ Corresponding author, myeongsup.lee@gmail.com, woonjusong@snu.ac.kr, martin.steinegger@snu.ac.kr

**Table of contents**

**Supplementary Figures**

Supplementary Figure 1. Purification of KYNases.

Supplementary Figure 2. PEGylation of KYNases.

Supplementary Figure 3. Taxonomic distribution of hits detected by search through UniProtKB and metagenomic databases.

Supplementary Figure 4. pLLM based regressor test on FPbase and FireProtDB by subsetting various size of training data.

Supplementary Figure 5. SeekRank results, ordered by MMseqs2 search result and predicted *k*_cat_/*K*_M_ values.

Supplementary Figure 6. Time-dependent consumption of L-KYN by Pf-K and K3.

Supplementary Figure 7. MD simulations for substrate binding.

Supplementary Figure 8. CD spectra of non-PEGylated and PEGylated Pf-K and K3.

Supplementary Figure 9. Antitumor effects of Pf-K-PEG and K3-PEG *in vivo*.

Supplementary Figure 10. The change of body weight during the PEG-KYNase treatment.

Supplementary Figure 11**.** Antitumor effect of PEG-KYNase in colon cancer mouse model (CT26).

**Supplementary Tables**

Supplementary Table 1. Statistics of the training data.

Supplementary Table 2. The amino acid sequences of kynureninases used in this study.

Supplementary Table 3. Top 50 sequences from RF predictor of *k*_cat_/*K*_M_.

Supplementary Table 4. Steady-state kinetic parameters of kynureninases used in this study.

Supplementary Table 5. Cavity volume in the active site of Pf-K and K3.

**Supplementary Figure 1.** Purification of KYNases. An elution profile of (**A**) HisTrap affinity chromatography and (**B**) size-exclusion chromatography. The elution of KYNases is marked with an asterisk in (A) and (B). (**C**) Representative UV-Vis absorption spectrum of Pf-K. The absorption at 430 nm region indicates the presence of PLP-cofactor. (**D**) Representative SDS-PAGE of purified KYNases.

**
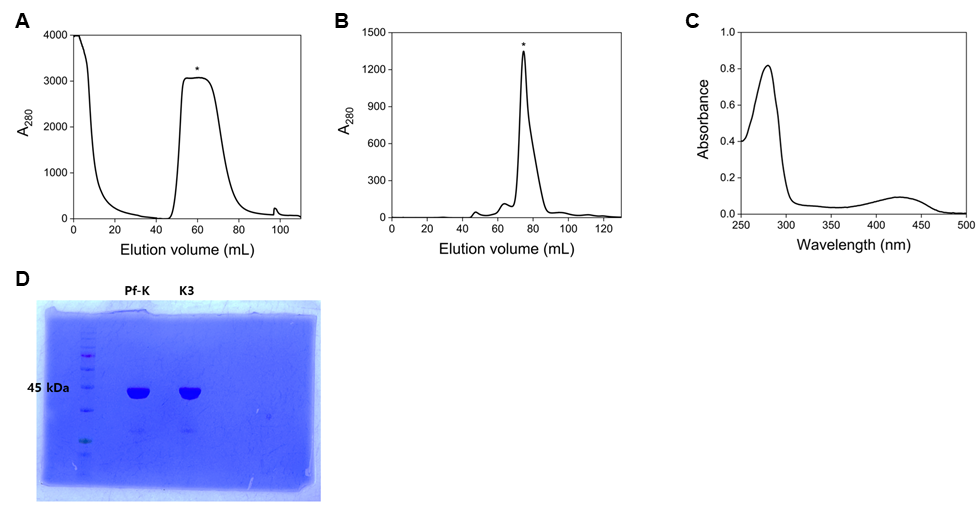
**

**Supplementary Figure 2.** PEGylation of KYNases. (**A**) FPLC chromatogram of non-PEGylated and PEGylated KYNases (**B**) Calculated molecular weight of KYNases before and after PEGylation using methoxy-PEG-CO(CH_2_)_2_COO-NHS (5 kDa).

**
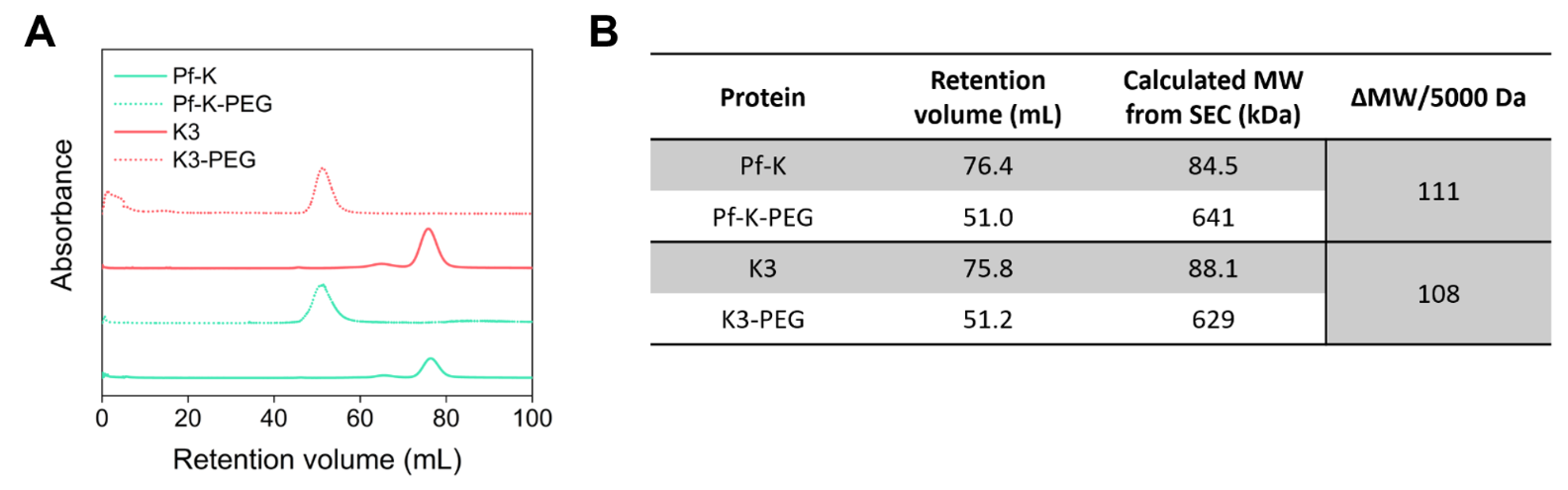
**

**Supplementary Figure 3.** Taxonomic distribution of hits detected by search through UniProtKB and metagenomic databases. Taxon were predicted by MMseqs2 taxonomy. Sankey plot was produced by Pavian (1).


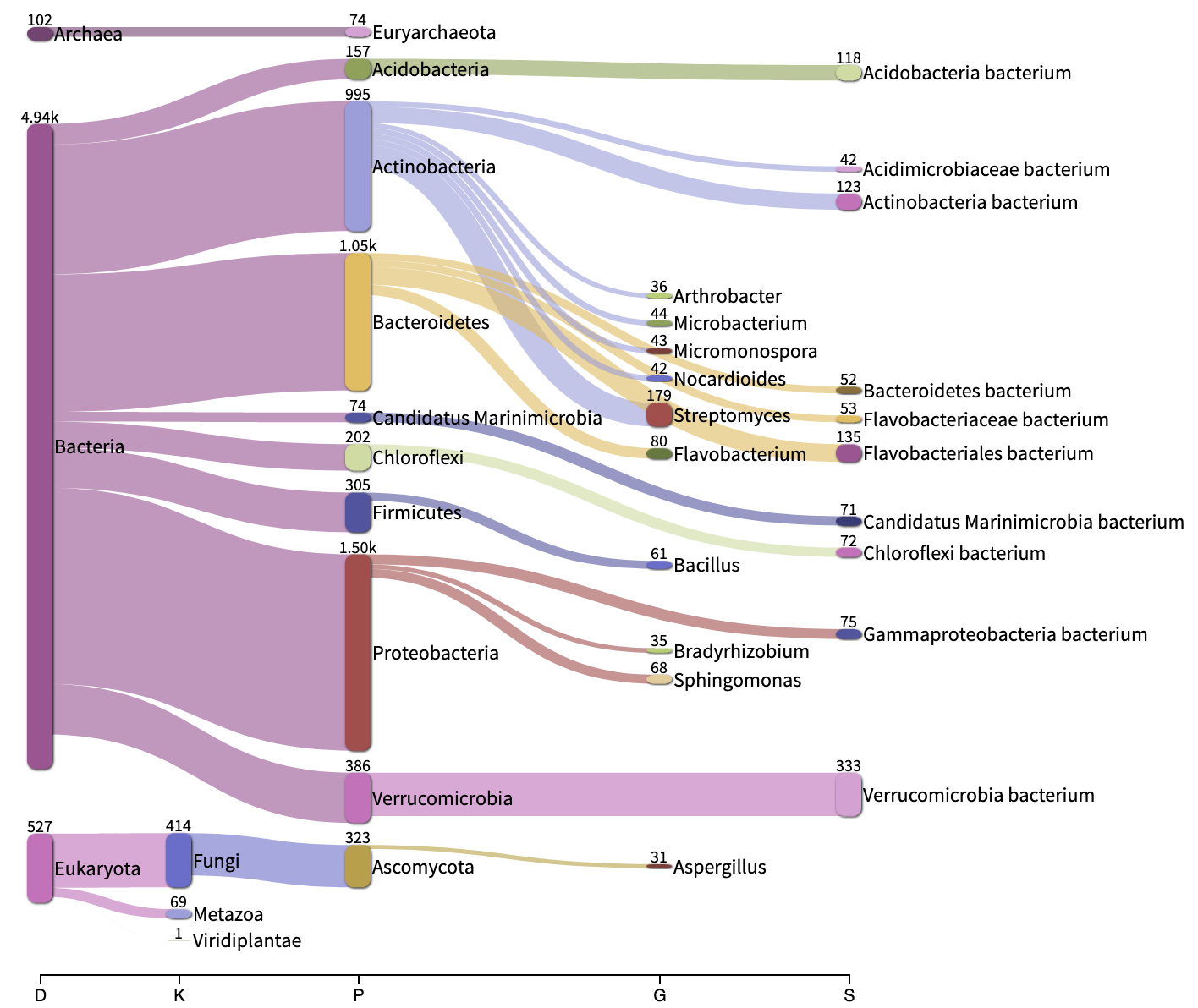


**Supplementary Figure 4.** pLLM based regressor test on FPbase and FireProtDB by subsetting various size of training data. (A) Scatter plots comparing actual and predicted values for brightness, emission peak wavelength, and melting temperature using the RF regressor trained on the entire dataset. The figure illustrates the predictive performance on the test set. (B) Spearman rank correlation between actual and predicted values as a function of sampled training data size for brightness, emission peak wavelength (from FPbase), and melting temperature (from FireProtDB). Predictions were made using k-nearest neighbors (KNN), random forest (RF), and support vector machine (SVM) regressors. The orange vertical line indicates the minimum dataset size required to achieve stable correlation results. (C) Comparison of Spearman rank correlations: between predictions from the RF and KNN regressors (black), and between RF predictions and actual values (gray). (D) Success rate of the RF regressor, showing the proportion of trials where the Spearman rank correlation exceeded 0.5.


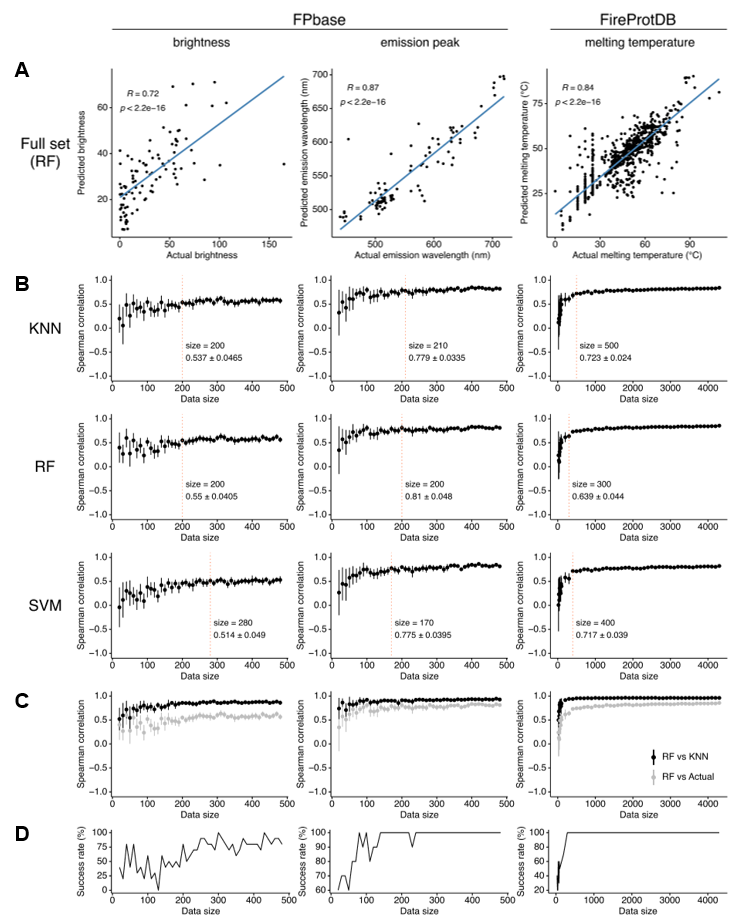


**Supplementary Figure 5.** SeekRank results, ordered by (A) MMseqs2 search result and (B) predicted *k*_cat_/*K*_M_ values. The left figures show all results and the right figures show the top 50 results. Pf-K, K1, K2, K3, and K31 are indicated with the dashed lines.


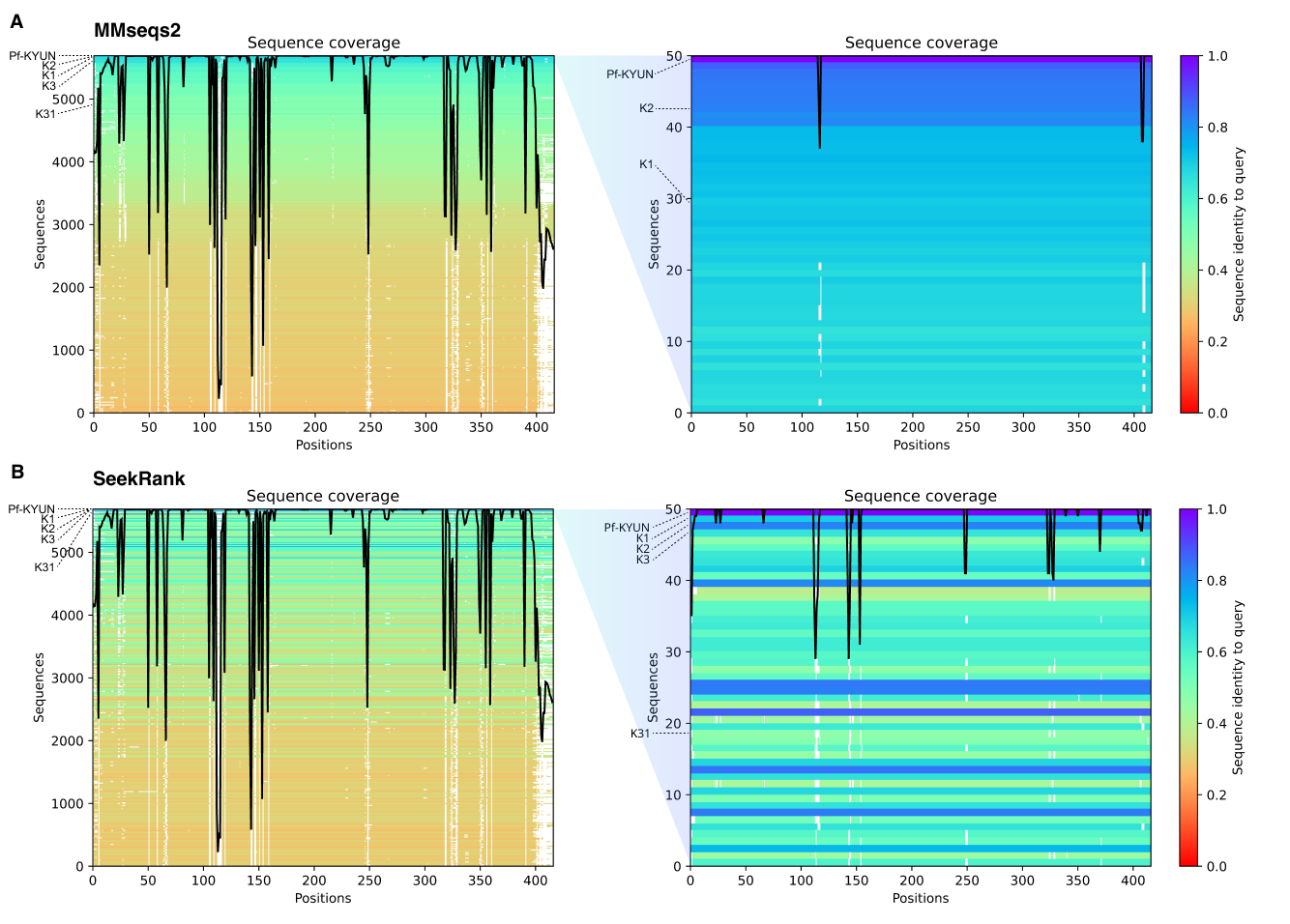


**Supplementary Figure 6.** Time-dependent consumption of L-KYN by Pf-K and K3. Time-dependent consumption of substrate (700 µM) was monitored by measuring absorbance (365 nm) at 37 °C. Control indicates the assay without the enzyme.


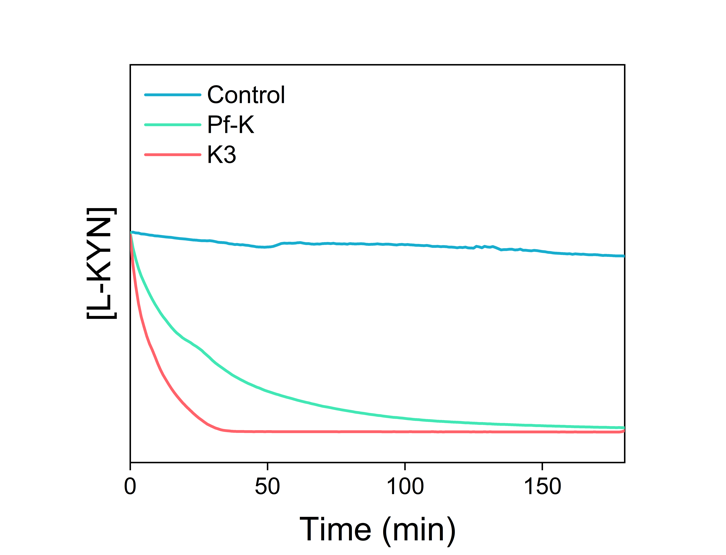


**Supplementary Figure 7.** MD simulations for substrate binding. (**A**) The dotted yellow line emphasizes L-KYN out of binding site due to high fluctuation. **(B)** The time-series of inter-atomic distances between the amine nitrogen of aniline ring of L-KYN and three residues from the active site of each monomer of K0. The asterisk marks (*) denote residues from the other symmetric monomer.


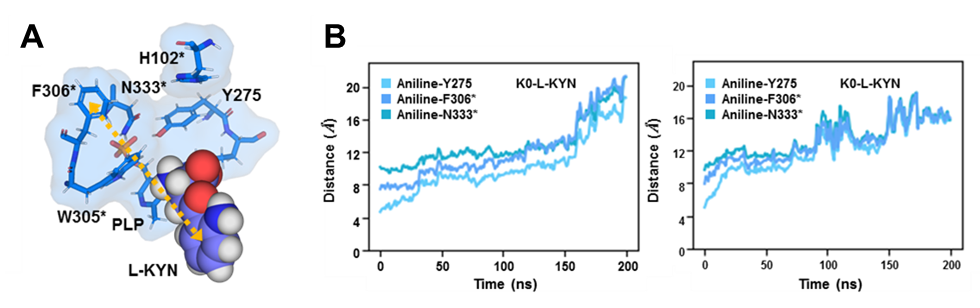


**Supplementary Figure 8.** CD spectra of non-PEGylated and PEGylated Pf-K and K3.


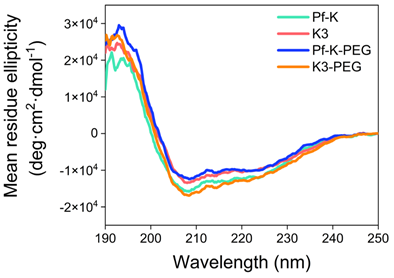


**Supplementary Figure 9.** Antitumor effects of Pf-K-PEG and K3-PEG *in vivo*. Tumor growth curve of B16-F10 bearing mice (C57BL/6) that were treated with peritumoral injection of control (PBS, n=13), Pf-K-PEG (20 mg/kg, n=13) and K3-PEG (20 mg/kg, n=13). Arrows indicate treatment point.

**
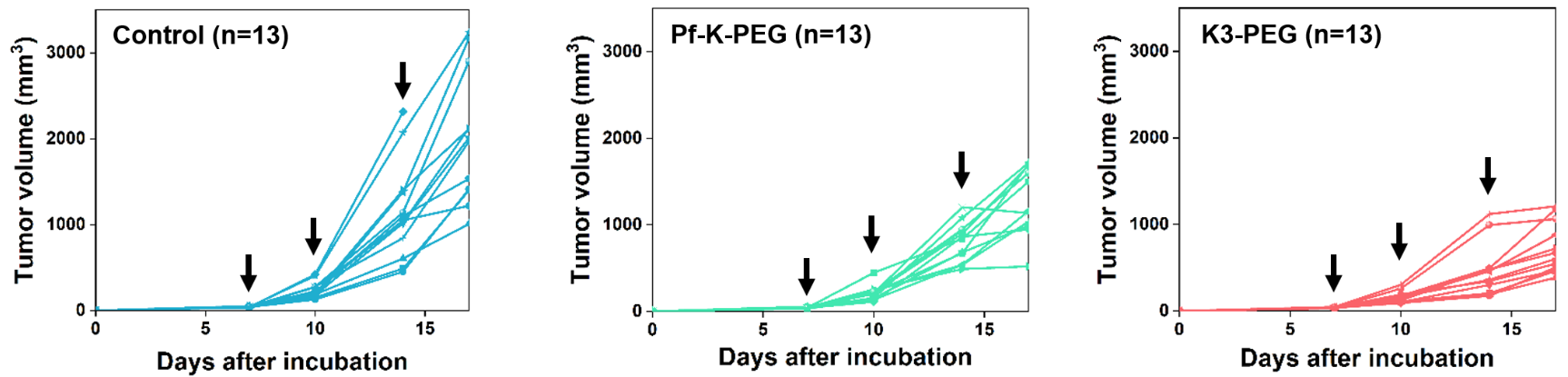
**

**Supplementary Figure 10.** The change of body weight during the PEG-KYNase treatment. B16-F10 bearing mice (C57BL/6) were treated with a peritumoral injection of control (PBS, n=13), Pf-K-PEG (20 mg/kg, n=13) or K3-PEG (20 mg/kg, n=13) twice a week (total 3 times) and the body weight was measured twice a week after treatment until the end of the experiment. There is no statistically significant difference between treatment groups.


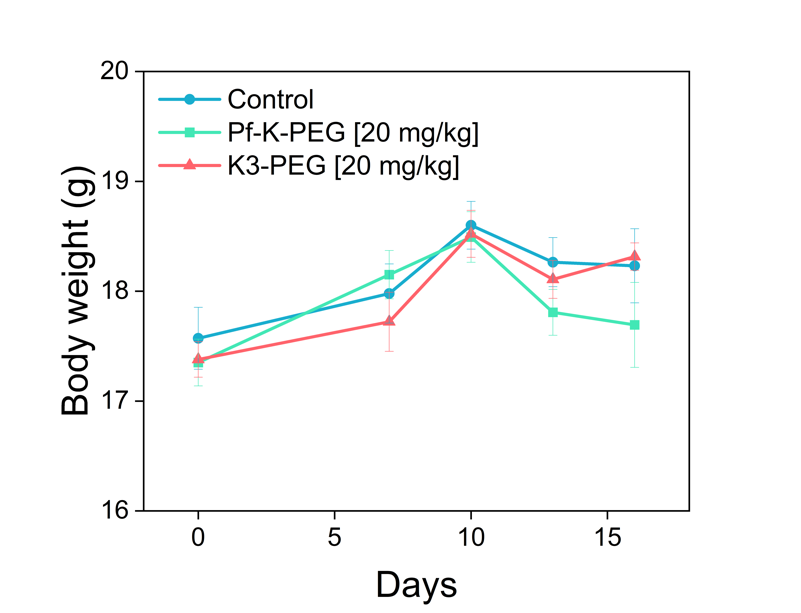


**Supplementary Figure 11.** Antitumor effect of PEG-KYNase in colon cancer mouse model (CT26). (**A**) Tumor growth curve of CT26 bearing mice (BALB/c) that were treated with a peritumoral administration of 20 mg/kg (vehicle, Pf-K-PEG or K3-PEG). (**B**) Mean and individual tumor growth curves over time. Statistical significance was verified using a two-tailed t-test (* *p* ≤ 0.05, ** *p* ≤ 0.01, *** *p* ≤ 0.001). Error bars represent the mean± SEM.


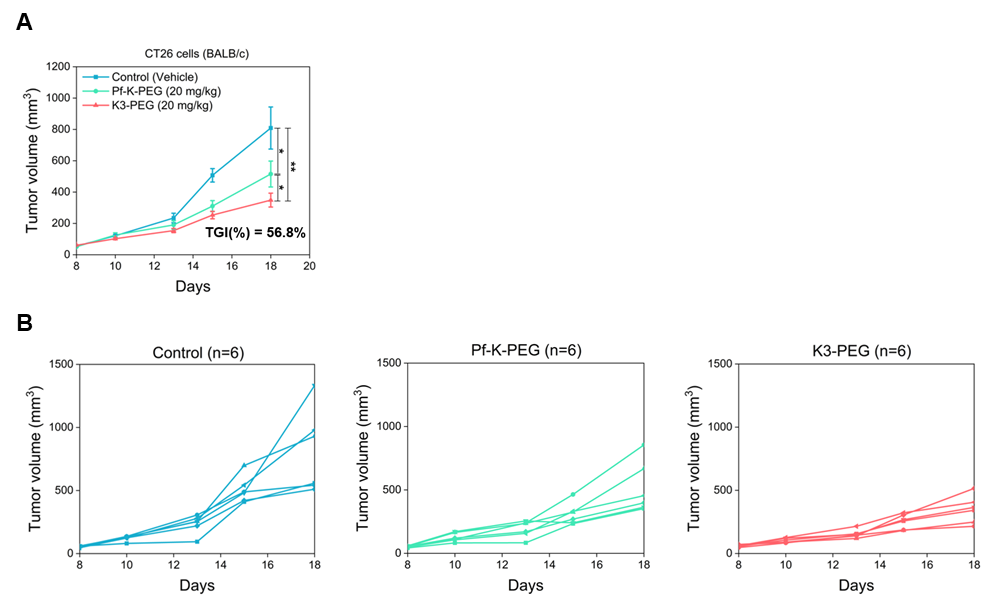


**Supplementary Table S1.** Statistics of the training data.

| Number of sequences | Sum of the sequence lengths | Minimum length of the sequences | Average length of the sequences | Maximum length of the sequences | Average pairwise sequence identity |
| --- | --- | --- | --- | --- | --- |
| 159 | 73,706 | 416 | 463,6 | 466 | 0.926 |

**Supplementary Table 2.** The amino acid sequences of kynureninases used in this study. The N-terminal His-tag was underlined.

| Protein | Amino acid sequence |
| --- | --- |
| Pf-K from *Pseudomonas fluorescence* | MGSSHHHHHHSSGLVPRGSHMTSRSHCQTLDAQDPLAPLRDQFALPAGVIYLDGNSLGARPVASLARAQQVIAEEWGNGLIRSWNSAGWADLSLRLGNRLAPLIGAGAGEVAITDTTSINLFKVLSAALTVQRQREPARKVIVSEASNFPTDLYIAEGLAELLQQGYCLRLVNSPDELPQAIDADVAVVMLTHVNYKTGYMYDMQALTALSHECGALSIWDLAHSAGAVPIDLRAAGADYAIGCTYKYLNGGPGSQAFVWVNPALVDQVRQPLSGWFGHTRQFAMESNYAPSAGIARYLCGTQPITSLAMVECGLQIFEQTDMACLRRKSLALTDLFIALVEARCAAHGLVLITPREHARRGSHVSFEHPEGYAVIQALIARGVIGDYREPRIMRFGFTPLYTRFSEVWDAVEILGEILDESTWDQPQFKVRHSVT |
| K1 from *Pseudomonas sp.* | MGSSHHHHHHSSGLVPRGSHMATQQHLRELDLADPLAALRDEFALPEGVIYLDGNSLGARPKAALARATQMIEQEWGEGLIRSWNTAQWSTLSARLGDKLAPLIGADSGEVVITDTTTVNLFKILAAGLRIQAERAPQRKIILSELHNFPADLYVIEGLADLLQQGYELRLIERPQDLPGLLDDQVALVLLTHVNYKSGHMYDMTATTGLIHQHGALALWDLAHSAGAVPVQLKAANADYAIGCTYKYLNGGPGSPAFVWVSPQLCDQVWQPLSGWWGHARQFAMEPHYAPASGITRYLCGTQPLVSLGMVESGLDIFAKTSMQALRNKSLALTDLFIELVEARCKDHPLTLITPRDHAQRGSHVSFEHPEGYAVVQALIARGVIGDYREPRIMRFGFTPLYTSFQDVGAAVQALVEVLDSQQWREPQFQTRHSVT |
| K2 from *Pseudomonas sp.* | MGSSHHHHHHSSGLVPRGSHMITRNDCLALDAQDPLAHLRHQFALPEGVIYLDGNSLGARPIAALERAQAVIAEEWGNGLIRSWNSAGWLDLPERLGNRLAGLIGAGEGEVVVTDTTSINLFKVLGAALRVQAMRAPTRRVIVSESSNFPTDLYIAEGLMDLLQQGYSLRLVDSPEELAQAIDQDTAVVMLTHVNYKTGYMHDMQAVTVLIHECGALAIWDLAHSAGAVPVDLRQAGADYAIGCTYKYLNGGPGSQAFVWVAPQLCDLVTQPLSGWFGHSRQFDMASGYEPSSGIARYLCGTQPITSLAMVECGLEIFAQTDMPSLRRKSLALTDLFIQMVEQRCAAHDLKLITPREHARRGSHVSFEHPQGYAVIQALIARGVIGDYREPRIMRFGFTPLYTSFTEVFDAVQILGEILDQQTWSQAQFQVRHSVT |
| K3 from *Bordetella sp.* | MGSSHHHHHHSSGLVPRGSHMHTREACLQADQQDPLAPLKAQFDIPAGVLYMDGNSLGVLPKAAVARSAQVIQQEWGQGLIRSWNDASWFELPSRLGDKLGRLIGAGTGQVVVTDTTSLNLFKSLAAAIRIQQQAAPQRKIIVSERDNFPTDLYMIQGMIDLLQQGYEMRLVDEDLSLEQALDDSVAVLLLSHVNYRTGHMYDMADVTAQAHARGALTIWDLAHAAGAVPVDLTGANADFAVGCTYKYLNGGPGAPAFIWVAPRHTDHFWQPLSGWWGHQRPFDMAVNYEPAGGIRRYLCGTQPIVSLSLVECGLDISLQADMNEVRRKSLALTDLFIALVESRCARHPLTLVTPREHAHRGSHVSLRHPHGYAVMQALIARGVIGDYREPEVLRFGFTPLYFGYTDVWDAVEILTDVLDSEIWKQPEFSRRGAVT |
| K31 from *Rheinheimera sp.* | MGSSHHHHHHSSGLVPRGSHMTCAALQQRDIDDPLSGKRAAFYLPDNTLYLDGNSLGAMPKIAAERAAEVVSQQWGEGLITSWNRHHWIDLPFSVGDKIGHLIGAAPGQVICCDSTSVNLFKVLCAALSLQPARSKVLSVSGNFPTDLYMVEGLSALTGNNHYQLQLVDESELEQAITGQVAVLLLTHVDFRSGRLFDMAKLTRLAQDKGALVIWDLAHSAGALPLALDQCHVDFAVGCGYKYLNGGPGAPAFLYAAKRHHAMLQQPLTGWMGHKTPFSFSTQYEKASGIAQFLTGTPPVISMSVLDAALDVFADVDIAQLRQKSLALSDCFHQLVSQNDCLNELERITPYAAAERGSQLAYRHPQAYALCQALIKQGVIADFRAPDILRLGFTPLYLRYIDVWTAVEILADVMRSSEYLKAEYQIKQKVT |

**Supplementary Table 3.** Top 50 sequences that RF predictor predicts having high *k*_cat_/*K*_M_ including the Pf-K which is used to search the homologs. Full table with sequence is available on zenodo ([10.5281/zenodo.10517668](https://zenodo.org/records/10517668)).

|  | **Sequence identifier** | **Per. Ident (pf)** | **Normalized Random Forest predictor** | **Taxonomical label** | | **Uniprot Taxonomical label** | |
| --- | --- | --- | --- | --- | --- | --- | --- |
| Pf-K | pf-KYNase (Nat. Biotechnol.) | 100 |  | Pseudomonas | fluorescence |  |  |
| K1 | A0A0Q5EY38 | 70.67 (294/416) | 1 | Pseudomonas | sp. | Pseudomonas | sp. |
| K2 | ETNmetMinimDraft_22_1059887.scaffolds.fasta_scaffold818137_1 | 82.45 (343) | 0.983639 | Pseudomonas | sp. |  |  |
| K3 | A0A261TMR2 | 64.42 (268) | 0.975792 | Bordetella | genomosp. | Bordetella | genomosp. |
|  | I8U8E1 | 48.28 (196) | 0.969635 | Alishewanella | agri | Alishewanella | agri |
|  | APFEC2959095171_1045051.scaffolds.fasta_scaffold03691_1 | 54.78 (235) | 0.969356 | Limnohabitans | sp. |  |  |
|  | A0A1Y6B7Q2 | 63.46 | 0.968624 | Pseudogulbenkiania | subflava | Pseudogulbenkiania | subflava |
|  | SRR5690554_2167168 | 65.4 | 0.966497 | Pusillimonas | sp. |  |  |
|  | A0A077LGT1 | 68.9 | 0.962143 | Pseudomonas | sp. | Pseudomonas | sp. |
|  | N9BME8 | 54.5 | 0.958279 | Acinetobacter | soli | Acinetobacter | soli |
|  | CryBogDrversion2_8_1035294.scaffolds.fasta_scaffold116296_2 | 81.9 | 0.957185 | Pseudomonas | sp. |  |  |
|  | YNPNPStandDraft_1061719.scaffolds.fasta_scaffold472067_1 | 39.8 | 0.956657 | Gammaproteobacteria | bacterium |  |  |
|  | EndMetStandDraft_7_1072992.scaffolds.fasta_scaffold491733_1 | 41.8 | 0.954957 | Alteromonadaceae | bacterium |  |  |
|  | A0A256CJ58 | 56.7 | 0.954855 | Ignatzschineria | sp. | Ignatzschineria | sp. |
|  | S3N7J3 | 56.5 | 0.953425 | Acinetobacter | rudis | Acinetobacter | rudis |
|  | AntAceMinimDraft_12_1070368.scaffolds.fasta_scaffold29590_2 | 60.1 | 0.953033 | Rhodoferax | sp. |  |  |
|  | A0A261QU68 | 62.7 | 0.952976 | Bordetella | genomosp. | Bordetella | genomosp. |
|  | A0A077KWD1 | 55.6 | 0.952306 | Acinetobacter | guillouiae | Acinetobacter | guillouiae |
|  | A9IDE5 | 62.2 | 0.950562 | Bordetella | petrii | Bordetella | petrii |
|  | A0A235G0T9 | 62 | 0.950301 | Rhodococcus | sp. | Rhodococcus | sp. |
|  | X2HAC8 | 57.4 | 0.948792 | Snodgrassella | alvi | Snodgrassella | alvi |
|  | A0A126ZI65 | 57.9 | 0.948713 | Variovorax | sp. | Variovorax | sp. |
|  | A0A136A5P8 | 47.4 | 0.947544 | Paraglaciecola | hydrolytica | Paraglaciecola | hydrolytica |
|  | A0A0N7J9K8 | 54.3 | 0.947399 | Limnohabitans | sp. | Limnohabitans | sp. |
|  | A0A1T1HHN4 | 84.1 | 0.947314 | Pseudomonas | sp. | Pseudomonas | sp. |
|  | A0A0S4HSW4 | 83.4 | 0.947045 | Pseudomonas | sp. | Pseudomonas | sp. |
|  | A0A1I1ZZ70 | 61.4 | 0.946658 | Acidovorax | wautersii | Acidovorax | wautersii |
|  | K6YC39 | 41.1 | 0.945445 | Aliiglaciecola | lipolytica | Aliiglaciecola | lipolytica |
|  | AP41_2_1055478.scaffolds.fasta_scaffold381214_1 | 87.7 | 0.944619 | Pseudomonas | gessardii |  |  |
|  | SRR5512138_1738479 | 43.4 | 0.942924 | Chloroflexi | bacterium |  |  |
|  | OM-RGC.v1.013517068 | 62.9 | 0.941772 | Paralcaligenes | ureilyticus |  |  |
| K31 | A0A117NJK5 | 47.00 (193) | 0.941642 | Rheinheimera | sp. | Rheinheimera | sp. |
|  | Laugrespbdmm15sd_2_1035082.scaffolds.fasta_scaffold306765_1 | 48.7 | 0.9411 | Limnohabitans | sp. |  |  |
|  | HotLakDrversion2_2_1075449.scaffolds.fasta_scaffold304163_2 | 56.9 | 0.940969 | Variovorax | sp. |  |  |
|  | A0A1E8CME8 | 45.2 | 0.940922 | Pseudohongiella | acticola | Pseudohongiella | acticola |
|  | A0A1F4M7D4 | 67.4 | 0.940767 | unclassified | Burkholderiales | Burkholderiales | bacterium |
|  | A0A1H3SQJ2 | 85 | 0.938853 | Pseudomonas | sp. | Pseudomonas | sp. |
|  | A0A254VHC2 | 65.6 | 0.936334 | Xenophilus | sp. |  |  |
|  | SaaInlV_150m_DNA_6_1039752.scaffolds.fasta_scaffold116048_1 | 42.2 | 0.935275 | Chloroflexi | bacterium |  |  |
|  | SaaInlV_130m_DNA_2_1039683.scaffolds.fasta_scaffold216743_1 | 67.4 | 0.933115 | unclassified |  |  |  |
|  | A0A285I0Z2 | 48.8 | 0.932615 | Rheinheimera | tuosuensis | Rheinheimera | tuosuensis |
|  | A0A1C6MKP5 | 64.9 | 0.930615 | Vogesella | sp. | Vogesella | sp. |
|  | A0A1H0XRK9 | 83.6 | 0.930334 | Pseudomonas | moorei | Pseudomonas | moorei |
|  | A0A2E5UZY5 | 52.8 | 0.929895 | Hyphomonadaceae |  | Oceanicaulis | Sp. |
|  | GraSoiStandDraft_41_1057321.scaffolds.fasta_scaffold1785920_2 | 64.8 | 0.929891 | Undibacterium | pigrum |  |  |
|  | RifCSPhighO2_02_1023873.scaffolds.fasta_scaffold28721_2 | 56.7 | 0.929262 | Burkholderiales | bacterium |  |  |
|  | EndMetStandDraft_2_1072991.scaffolds.fasta_scaffold155171_3 | 53.9 | 0.92926 | Limnohabitans | sp. |  |  |
|  | V5Z4I3 | 74 | 0.9292 | Erwinia | piriflorinigrans | Erwinia | piriflorinigrans |
|  | A0A094J787 | 45.8 | 0.928889 | Idiomarina | atlantica | Pseudidiomarina | atlantica |
|  | APSaa5957512622_1039677.scaffolds.fasta_scaffold910177_1 | 57.5 | 0.928883 | Rhodoferax | sp. |  |  |
|  | OrbTmetagenome_4_1107371.scaffolds.fasta_scaffold01457_3 | 57.8 | 0.927544 | Hydrogenophaga | sp. |  |  |

**Supplementary Table 4.** Steady-state kinetic parameters of kynureninases used in this study. The activities were measured in triplicate. Standard deviations are shown in parentheses.

|  | *Identifier* | *k*_cat_ (s^-1^) | *K*_M_ (μM) | *k*_cat_/*K*_M_ (M^-1^ s^-1^) |
| --- | --- | --- | --- | --- |
| Pf-K | WP_017531066.1 (GenBank) | 9.0(1) | 78(3) | 11(0) $\times$ 10^4^ |
| K1 | A0A0Q5EY38 (Uniprot) | 8.3(6) | 185(38) | 4.5(10) $\times$ 10^4^ |
| K2 | Marine metagenome | 7.3(2) | 83(8) | 8.8(9) $\times$ 10^4^ |
| K3 | A0A261TMR2 (Uniprot) | 16(1) | 133(23) | 12(2) $\times$ 10^4^ |
| K31 | A0A117NJK5 (Uniprot) | 6.2(2) | 91(11) | 6.8(9) $\times$ 10^4^ |

**Supplementary Table 5.** Cavity volume in the active site of Pf-K* and K3. KYNases showing the van der Waals interactions with 3-OH-KYN versus L-KYN. The time-averaged values and standard errors for 1,000 snapshots with separate values provided for each symmetry-related monomer 1 and 2.

|  | Monomer 1 [Å3] | Monomer 2 [Å3] |
| --- | --- | --- |
| Pf-K*/3-OH-KYN | 2.4 *±* 0.2 | 2.6 *±* 0.2 |
| Pf-K*/L-KYN | 2.4 *±* 0.1 | 2.3 *±* 0.1 |
|  | Monomer 1 [Å3] | Monomer 2 [Å3] |
| K3/3-OH-KYN | 1.8 *±* 0.1 | 1.8 *±* 0.2 |
| K3/L-KYN | 1.8 *±* 0.1 | 2.0 *±* 0.1 |

**Reference**

1. Breitwieser, F.P. and Salzberg, S.L. (2020) Pavian: interactive analysis of metagenomics data for microbiome studies and pathogen identification. *Bioinformatics*, **36**, 1303-1304.
